# Empyema

**Published:** 1892-11

**Authors:** P. O’Keef

**Affiliations:** Oconto, Wis.


### EMPYEMA.*

By P. O'KEEF, M. D.,
OCONTO, WIS.

Empyema has been treated surgically since the days of Hippocrates. He and his disciples removed the pus by means of incision into the pleural cavity between the ribs and by trephining through a rib. Before the period of antiseptic surgery a

*Read before the Brainard Medical Society.

great many surgeons removed the pus by means of the trocar and canula, but as all could not be removed by these means on account of inability of the lung to expand and fill the chest, thus forcing out the pus, the operation had to be repeated, in some cases many times, to the great annoyance of the patient. In many cases the pus became ichorous, septic, and death resulted. This change in the character of the pus was supposed to be due to the entrance of air through the trocar. It was doubtless due to the entrance of septic material. After the invention of the aspirator, this instrument was hailed as the means long sought for, and tried in a number of cases, but with no better results so far as effecting cures were concerned than the trocar gave.

In fact it added one great danger. The suction made with the instrument in order to pump out all the fluid caused a number of sudden deaths, probably due to œdema of the sound lung. This complication necessitated the removal of only a small quantity of pus at a sitting and left us just where we were left by the trocar, and we were obliged to revert to the ancient method of treatment by free evacuation of the pus with additional antiseptic irrigation of the cavity. In fact an empyema is treated as an abscess, and free drainage and frequent antiseptic irrigation should be employed. I think it a good plan in all cases to resect a portion of a rib. The operation is quickly and easily done, and you can insure permanent free drainage. There will be nothing to compress the rubber tubing which is generally used and you can remove clots which would not pass through an incision between the ribs and whose presence you would not suspect unless you could explore with the finger.

The difficulty with an incision without rib resection is the impossibility of obtaining permanent free drainage. In a few days the ribs fall in and overlap and cause such an amount of pressure on the drainage tubes as to necessitate resection in the majority of cases.

I have another potent reason for urging resection, and that is because it aids nature materially in obliterating the cavity. As soon as the pleural cavity is emptied the lung begins to expand, and if it is not too long compressed and bound down by granulation tissue it will soon fill the cavity. At the same time, the ribs fall in and overlap each other and thus decrease the size of the cavity particularly if a section of one of the ribs is removed. If the lung expands so as to fill the chest we soon have a cure without any deformity. But in many cases the lung expands so slowly that a curved spine and a flattened chest is the result. In still other cases there does not seem to be the least expansion of lung and the ribs cannot possibly fall in sufficiently to obliterate the cavity. Here again the surgeon can greatly aid by resecting a portion of several ribs, thus allowing the chest wall to fall against the compressed lung and we soon have obliteration of the cavity which is the one thing needed ; for so long as a space exists between the lung and the chest wall pus will be formed. In order to induce rapid expansion of the compressed lung, and, at the same time, to carry out in the most perfect manner antiseptic treatment of the cavity, I have devised a scheme, which for want of a better name I call *syphonage*.

I first resect about one to one and one-half inches of the fifth or sixth rib subperiosteally, then as large an opening as this will permit is made into the pleural cavity. A finger is introduced to explore for clots, and if any are found they are removed and the cavity thoroughly irrigated with salicylic or boric acid solution. I now introduce two rubber tubes one ten inches long, ⅛ inch tubing, for conveying the antiseptic fluid to the bottom of the cavity. The other tube should be four or five feet long, ¼ inch tubing. Two or three lateral holes are made within one inch of the end of this piece, and this end and one end of the small tube are fastened with a couple of stitches of silk. These ends are now introduced to the bottom of the cavity and held by an assistant.

I now suture the wound close up to the tubes and apply a large layer of absorbent cotton saturated with balsam of Peru over the wound and around and between the tubes. This part should be, when packed, ½ inch thick and four or five inches broad. This is covered with a thick layer of dry absorbent cotton packed firmly around the tubes and pressed against the chest. A large safety pin is now passed through both tubes in such a way as not to obstruct them, and as close to the cotton pad as possible. Next a piece of rubber adhesive plaster six inches wide and long enough to reach two-thirds around the chest is applied, the two tubes being drawn through a slit two inches long in its center. This holds the cotton firmly against the chest preventing entrance of air and by means of the safety pin holds the tubes firmly in position, and the wound is antiseptically dressed.

The patient should now be put into a bed two to two and one-half feet high, the end of the long tube with weight fastened to it dropped into a jar of carbolized water and the nozzle of the irrigator introduced into the smaller tube and the cavity again irrigated. Holding the large tube near the chest a little above the patient you insure the complete filling of the cavity with water and expulsion of all the air which has entered. As soon as the irrigation ceases the water is syphoned out of the cavity until an equilibrium is established.

At each expiration and when coughing more water is forced out and in being drawn back into the cavity will exert a traction of about one pound to every square inch of lung surface, and as this is continuous it must have a powerful influence in expanding the lung.

This dressing should not be disturbed for eight or ten days, but the cavity should be irrigated once or twice a day and a fresh solution put into the jar after each dressing, and on removal of the nozzle of the irrigating tube care should be taken to avoid the entrance of air, and the rubber tube should be corked immediately. In a few days, if the patient wishes to sit up, the end of the tube can be put in a rubber bottle containing the solution, and he can carry it about with him if he wishes to move. I have during the last year treated three cases on the plan just described with results as follows :

CASE I.—H. B., aged 20 years, came to my hospital June 2, 1891. Had pneumonia in February, for which I attended him. Did not see him again until he came on June 2d. Found right side of chest bulging; respiration 40; pulse 110; temperature 102°. I diagnosed empyema, which was verified by hypodermic needle. I immediately removed 1½ inches of fifth rib between axillary and mammary line, and made an incision into the pleural cavity. A large quantity of pus escaped, and the lung could not be reached with the finger in any direction. The cavity was thoroughly washed out and treated on the plan already described, with the result that in eighteen days the tubes were removed.

The patient left the hospital at the end of the fourth week, the sinus completely healed.

CASE 2.—On June 22d, 1891, I was called by Dr. Oswaldt, of Stiles, to operate for empyema. R. V., aged 14 years, said he was struck by a plow handle in the right side about six weeks ago. Respiration short and rapid; pulse 130; temperature 104°; very weak and amaciated. I removed one and one-half inches of the fifth rib as in Case 1, and on introducing my finger found a clot the size of my fist, which I removed. The cavity was irrigated and dressed as described, and left in care of Dr. Oswaldt. As the patient lived nine miles from the doctor irrigation was done only every other day, but the carbolized water was changed twice a day and the wound entirely healed in six weeks. In this case the lung was completely compressed, as there was at least two quarts of pus. The empyema was probably due to a fracture of a rib.

CASE 3.—L. D., aged 4 years. Empyema right side following catarrhal pneumonia about the first of December, 1891. On January 10, 1892, I removed one inch of fifth rib as in Cases 1 and 2. One week later the lung was completely expanded. I drew tubes one inch each day, and on the tenth day removed them entirely. Sinus healed in a few days.

In contrast with these cases here is one I treated in 1889:

CASE 4.—Barney F., a boy in my employ complained one night of not feeling well, being chilly, etc. He had pains all over his body. Appetite had been poor for about a week. I gave him a dose of quinine and told him he would feel better in the morning. Was called up about four o'clock next morning. I went up to see Barney before going out. I found him cyanosed. I put my ear to his chest and found the heart to the right of the sternum. The left side was completely dull. I gave him whiskey and used the trocar at once and removed about two quarts of pus. The next day I made an incision between the ribs and introduced a rubber drain and irrigated every day.

About three weeks later the ribs had so overlapped as to compress the tube, and I inserted a metal one which answered very well.

I wanted to resect some of the ribs to obliterate the cavity, but was not allowed, so he is wearing the tube yet.

In this case the lung could not have been compressed but a short time, and with a little assistance by traction I think the result would have been better.

